# Knock-Knees

**Published:** 1888-10-13

**Authors:** 


### Within the Wards.

### KNOCK-KNEES.

There are few modern operations in surgery which appeal more to the general and professional imagination than those performed for the cure of what is called "knock-knee." The deformity in question, at all times unsightly, is sometimes positively repulsive. Mr. A. G. Miller, of Edinburgh, publishes, with two illustrations, a remarkable case in the first number of the *Illustrated Medical News.* The case is that of a girl of sixteen, whose left knee, before operation, lay upon the right in the standing posture, and in immediate contact with it, whilst the feet sprawled more than half-a-yard apart. The angle formed at each knee was almost a right angle. Two operations were required for each leg. The first consisted in excising a wedge-shaped piece of bone from the tibia, below the knee-joint, the second in excising a similar wedge-shaped piece from the femur above the knee-joint, the joint itself not being interfered with. By these operations both legs were made comparatively straight, and walking was rendered easy. Simple as this method seems, and simple as it is, at any rate in description, it is entirely modern. There may still be seen among middle-aged persons numbers who can neither walk nor stand. In future such deformed people will only have themselves to thank if they are not cured and made like ordinary human beings.

